# Urine as a Main Effector in Urological Tissue Engineering—A Double-Edged Sword

**DOI:** 10.3390/cells9030538

**Published:** 2020-02-26

**Authors:** Tariq O. Abbas, Tayyiba A. Ali, Shahab Uddin

**Affiliations:** 1 Laboratory for Stem Cell Research, Department of Health Science and Technology, Aalborg University, 9220 Aalborg, Denmark; 2 Pediatric Urology Section, Sidra Medicine, Doha 26999, Qatar; 3 College of Medicine, Qatar University, Doha 2713, Qatar; 4 Surgery Department, Weill Cornell Medicine—Qatar, Doha 24144, Qatar; 5 Translational Research Institute, Academic Health System, Hamad Medical Corporation, Doha 3050, Qatar; tayyiba1991@gmail.com (T.A.A.); skhan34@hamad.qa (S.U.)

**Keywords:** urethra, tissue engineering, cytotoxicity, urinary reconstruction, urothelial cells

## Abstract

In order to reconstruct injured urinary tract tissues, biodegradable scaffolds with autologous seeded cells are explored in this work. However, when cells are obtained via biopsy from individuals who have damaged organs due to infection, congenital disorders, or cancer, this can result in unhealthy engineered cells and donor site morbidity. Thus, neo-organ construction through an alternative cell source might be useful. Significant advancements in the isolation and utilization of urine-derived stem cells have provided opportunities for this less invasive, limitless, and versatile source of cells to be employed in urologic tissue-engineered replacement. These cells have a high potential to differentiate into urothelial and smooth muscle cells. However, urinary tract reconstruction via tissue engineering is peculiar as it takes place in a milieu of urine that imposes certain risks on the implanted cells and scaffolds as a result of the highly cytotoxic nature of urine and its detrimental effect on both growth and differentiation of these cells. Both of these projections should be tackled thoughtfully when designing a suitable approach for repairing urinary tract defects and applying the needful precautions is vital.

## 1. Clinical Need

The urinary tract transports urine and stores it after kidney filtration. The urinary bladder is the principal reservoir for urine while both ureters and urethra play the role of passageways. The unique feature of bladder repetitive contraction and expansion, together with a sound barrier of urine and potency to bear the pressure of urine make the bladder a complex organ. On the other hand, urethra and ureters are less complex but are still under radial pressure and shear strain of fluid during the transit of urine. The walls of the lower urinary tract are lined by urothelium, a multilayered epithelial lining which protects stroma against urine leak [1,2].

Several congenital and acquired pathologies can affect the human urinary tract, such as hypospadias, strictures, fistulas, trauma, and cancer. As a result, various reconstructive approaches have been utilized to restore the function of the urinary tract in these circumstances. However, certain factors make this process very complicated for surgeons, including poor quality of local tissues, which would mandate extra tissues for replacement. Additional sources of tissues, among others, include genital skin [3], buccal mucosa [4], and lingual mucosa [5]. However, significant complications, donor site morbidity, and limited tissue quantities that can be obtained from these sources restrict their utilization and provide opportunities for alternative approaches, including tissue engineering [6]. Healthy urethra also works as a urine leakage barrier throughout micturition [7] and serves as a conduit for an adequate antegrade passage of seminal fluid in adult men during sexual intercourse [8]. The urethra has unique mechanical characteristics that arise from its elastic elements and smooth muscles, providing exceptional compliance that enables urine to pass under low resistance during voiding [9]. For the application of urological tissue engineering, several synthetic and natural biodegradable scaffolds, either acellular or cell-seeded, have been studied [10]. Scaffolds provide structural support and guide platforms for differentiated stem cells or mature cells to promote tissue regrowth. It has been shown that the matrices of cell-seeded collagen formed healthy urethra tissues, whereas collagen matrices with no cells seeded showed reduced tissue formation [11].

If the scaffolds are pre-seeded with cells, adverse outcomes such as graft shrinkage could potentially be avoided. However, additional challenging and time-consuming measures are required for harvesting, isolating, and seeding cells on scaffolds in order to seed such scaffolds [12]. Implantation of acellular constructs directly for the reconstruction of bladder cause graft shrinkage, which can be alleviated by pre-seeding cells on scaffolds. If a suboptimal tissue engineering approach is used without taking into consideration specific cellular functions and features, such as properties of biochemical urinary organs, in the regeneration of urothelium, there would be a risk of tissue fibrosis and formation of stones, while insufficient properties of urine barrier can develop in the regenerated tissues. Moreover, the long-term biological effect of such methodologies is not yet entirely understood [13].

## 2. Urothelium as a Urine Barrier

Urothelium—a specialized multilayer epithelium that lines the bladder, ureter, and urethra—provides a tight barrier for urine [13]. Normal urothelium is highly specific and has evolved to function in a dynamic biochemical and mechanical setting, where it contracts and stretches repetitively. The primary role of urothelium is to provide a barrier to solutes and ions present in urine and the release of chemical mediators and sensations (chemical, thermal, and mechanical sensors) [14].

The urothelium barrier is maintained through three structures, namely tight junctions situated between the superficial umbrella cells [15], uroplakin proteins in the membrane of the apical cell, and proteoglycans and urothelial glycosaminoglycan (GAG) that covers the umbrella cells. In order to prevent the formation of urinary tract stricture, the degeneration and formation of healthy urothelium containing these three structures is considered critical. Furthermore, overactivity of the bladder detrusor muscle, fibrosis, and inflammation are prevented by an intact urothelium regrowth [16]. Hence, if the urothelium is compromised, it can lead to many urologic pathologies, such as bladder cancer, urethral stricture, overactive bladder, recurrent urinary tract infection, and interstitial cystitis. Available evidence indicates that urothelial cells are more resistant to urine as compared to underling smooth muscle cells. In all cell lines, the toxic effect of urine has been confirmed by a real-time analysis, and simple urine addition has been shown to result in a fast decline in cell proliferation [17]. In comparison to Urothelial Cells (UCs) that were substantially resistant, adipose-derived stromal cells (ADSCs) are the most sensitive cell type [17].

When the shielding barrier of the uroplakin is dysfunctional, as is the case in the early phases of either urothelium regeneration or healing, agents that are cytotoxic bind directly to the anionic setting of underlying layers. Thus, a substantial impact of deeply penetrating urine on the implanted stem cells’ viability could take place [16]. Since the effect of urine is harmful to cellular components of the tissue-engineered urethral grafts, it is vital that scaffolds have sufficient impermeability. Moreover, it was recently shown that interstitial fluid flow plays a significant role in the wound healing response, implying that any leakage of urine from within the urinary tract may provoke surrounding fibrosis and lead to contracture [16]. Although scaffolds with low porosity levels have excellent potential resistance towards the penetration of fluid and toxic solutes of urine, they may inhibit cell migration within these scaffolds [18,19].

## 3. Bioengineered Urothelium

Bioengineered urothelium would present a valuable tool for urethral replacement, as it is resistant to the toxic effects of urine [17,20]. Presently, limited in vitro models of urothelium exist for different urologic diseases within the lower urinary tract to be treated by medications. Consequently, there is a unique need for engineered urothelium. In vitro approaches that have been developed to grow urothelial cells are considered unproductive, as they last several days and yield only a few additional passages, thus posing a considerable time and financial constraints on high-quality experiments aimed at elucidating their growth mechanism, differentiation, and proliferation. Although much progress has been made in order to understand the response of these cells to infection and wounds, functional urothelium regeneration remains a crucial challenge in urinary tissue engineering [21,22].

It has been determined in many studies that urothelial cells resulting from adult stem cells show a sufficient quantity of markers of urothelium but often lack proper histological structure and functional barrier capacity [23,24]. However, due to the absence of some critical markers, such as uroplakin III on the apical surface and absence of tight junctions, it is understood that substituted cells may form an inferior epithelial barrier.

The required in vitro urothelium cell expansion can be achieved by finding stem cell or progenitor cell sources that can regenerate, differentiate, and self-renew in situ. In this regard, it was determined that urothelial stem cells originate from upper, intermediate, or basal layers of the urinary tract as urine-derived stem cells [25]. Urothelial differentiation of UDSC offers a practically unlimited source of cells for tissue engineering. Various types of stem cells, including Mesenchymal Stem Cells (MSC) [24], Embryonic Stem Cells (ESC) [26], and induced pluripotent stem cell (iPSC) [27], have been explored towards urothelial cell differentiation. ESC or iPSC can produce functional urothelial cells; however, the differentiation process is time-consuming and prohibitively expensive. Moreover, when this process is applied, there is a risk that rare, undifferentiated cells may lead to the formation of teratoma.

On the other hand, MSCs give rise to a small number of urothelial cells with less pronounced differentiation ability than ESC, and typically in the same germ layer lineage, leading to extraordinary challenges [28]. For the purpose of urinary tract repair, functional smooth muscle, endothelial, peripheral and urothelial neurocytes with high efficacy would be the best source of stem cells that would be able to differentiate, permit collection through simple, low-cost, non-invasive and safe method; available universally or off-the-shelf, and able to produce organ-specific or tissue-specific stem cells from the urinary tract. Although it is well known that some types of cells are more favorable than others, it is undetermined whether a perfect stem cell with these abilities exists. The most commonly used MSCs are ADSCs, whereas BMSCs have several limitations in tissue engineering applications, including short in vitro duration (<10 passages), low capacity of differentiation (<5% for urothelial cells of endodermal lineage) and the need for invasive collection procedures [29].

## 4. Urine-Derived Stem Cells (UDSCs)

Urine-derived stem cells (UDSCs) can be harvested through non-invasive procedures [30] and sources including voided urine [22]. In order to use UDSCs successfully in tissue engineering approaches aimed at the reconstruction of the lower urinary tract, UDSC differentiation should be targeted towards three varieties of cells: urothelial cells, smooth muscles, and endothelial cells. Since stem cells possess remarkable regeneration capacity, many damaged tissues can be replaced by functional stem cells to restore normal tissue function [31]. It has been reported that by collecting fresh urine samples, the success rate of isolating and cultivating human urine-derived cells (HUDCs) can be improved. However, UDSC viability can be lost if the time of exposure is greater than 5 h, as pH will start to change and nutrients in urine will be lost [32].

### 4.1. Origin of Urine-Derived Stem Cells

UDSCs could be obtained from different species, including humans, as well as animals like monkeys, rabbits, and pigs. The exact sources of UDSCs will depend on the tissue supplier. In the kidneys, papilla or the renal tubules are the endogenous sources of stem cells. In glomeruli, the stem cells are obtained from glomerular parietal epithelial cells. By losing cell polarity, epithelial cells become mesenchymal stem cells through a process called epithelial-mesenchymal transition (EMT) [33]. These mesenchymal stem cells are multipotent and can be used in kidney regeneration and repair. These cells are characterized by high telomerase activity in comparison to other MSC types, making their proliferation capacity higher [31].

It has been shown that CD146þ/CD31 expression by UDSCs is similar to the expression of the same markers by podocytes and parietal cells in the glomerulus. On the other hand, epithelial cells of renal tubules, ureter, and bladder smooth muscle and urothelial cells do not exhibit a similar expression as UDSCs. These results suggest that UDSCs might be the transitional cells at the parietal cell/podocyte interface arising from kidney tissue [28].

Nevertheless, UDSCs should not be used in patients with bladder cancer because they could bear malignant cells. To overcome this hindrance, Bharadwaj et al. [34] have revealed that cells isolated from the upper urinary tract (UTCs) have characteristics similar to those of UDSCs. Furthermore, Chun et al. [35] pointed out that UTCs of patients with bladder cancer had no chromosomal anomaly or tumorigenicity in vivo. Accordingly, UTCs could be a beneficial cell origin for urologic tissue engineering in patients with bladder cancer.

### 4.2. Advantages of UDSCs

There are several advantages of human UDSCs relative to other stem cells. UDSCs and ADSCs were compared in a study conducted by Kang et al. [36] As a part of this investigation, UDSCs and ADSCs were collected from patients that required laparoscopic renal surgery. For each UDSC and ADSC type, colony formation, cell surface markers, cell proliferation, chromosome stability, immune modulation, and multi-lineage differentiation were investigated. In comparison to ADSCs, UDSCs showed an increased rate of cell proliferation, greater ability to form colonies, stronger positivity for stem cell marker expression, and increased efficiency for inhibition of immune cell activation [36]. Moreover, UDSCs showed a higher rate of myogenic, endogenic, and neurogenic differentiation. UDSCs have many other advantages, such as non-invasive harvest, low-cost, and straightforward procedures that can be used for the collection of cells. In contrast, liposuction and other invasive techniques are required for the isolation of MSCs, ADSCs, or hair-follicle stem cells (HFSCs) samples [37]. Furthermore, UDSCs (excluding UTI and anuria) can be obtained irrespective of the age, sex, and health status of an individual. Enzyme digestion is not required to sequester pure stem cells, while UDSCs exhibit telomerase activity, and they differentiate with higher efficacy into smooth muscle, urothelial, and endothelial cells. Table 1 details the advantages and characteristics of UDSCs in comparison to other SCs utilized in urological regenerative medical purposes.

#### 4.2.1. Cell Proliferation and Differentiation

UDSCs show a higher rate of myogenic, endogenic, and neurogenic differentiation and a lower osteogenic, adipogenic, and chondrogenic differentiation rate in comparison to ADSCs. Therefore, UDSCs instead of ADSCs can be used as an autologous stem cell source for muscle, neuron, and endothelial tissue reconstruction [36]. Available evidence indicates that UDSCs have efficient cappacity of multipotent differentiation into cells of cartilage, bone, muscles, and fat [42]. Hence, UDSCs are a viable source of cells for the treatment of erectile and renal dysfunction and various diseases, as well as bladder tissue engineering.

Urothelial differentiation of UDSCs presents an almost limitless cell source for tissue engineering or model fabrication with simple methods that can be used to isolate and expand urine cells. Additionally, the possibility of producing cells from urine samples makes the human urine-derived cells a smart choice for cell therapy [52]. This can produce high-quality cells, which can be expanded extensively [42]. Approximately 140 clones of UDSCs in 24-h urine collection from a single healthy individual can be attained [39]. Hence, 24-h urine output can yield > 1 × 10^8^ cells over three passages. This number is adequate for most of the intended applications. Moreover, as enzymes like collagenase are not needed for cell dissociation, cell viability during isolation is preserved [34,39]. In addition, numerous in vivo studies have failed to provide any evidence in human UDSCs for the potential oncogenic effect [38,51]. UDSCs are innate to the urinary tract, and with urine contact, they can survive similarly as healthy urothelial cells.

It has been shown that cell subpopulation isolated from urine could differentiate into numerous bladder cell lineages and exhibit features of progenitor cells. In an experiment conducted by Zhang Y et al. [40], three phenotypes were shown by isolated cells, i.e., progenitor-like cells, differentiating, and completely differentiated. Cells that were single progenitor could differentiate into the lineages of cells that express smooth muscle, urothelial, interstitial, and endothelial cell markers. Hence, cells derived from urine can be used as an alternative source for tissue engineering and urinary tract reconstruction.

The urothelial differentiation was shown to induce the expression of uroplakin-III, the anion exchange proteins AE3 and AE1, cytokeratin (CK) 7, and myosin, desmin, and smoothelin [38]. Additionally, when co-culturing of urothelial differentiated UDSCs and myogenically-differentiated UDSCs was done on decellularized collagen matrix-like SIS or bladder submucosa, UDSC in vitro structure was similar to that of the bladder wall [40,53].

Epithelialization process of graft implantation takes almost seven days following the augmentation of the urinary tract wall. Urothelium starts as a monolayer for the complete coverage of the luminal graft surface. It then gradually transforms into normal multilayered urothelium not distinguishable from healthy surrounding tissues. Urothelium impermeability is highly dependent on cell-cell interactions [54]. Thus, the formation of tight cell-cell interactions on the luminal surface of a scaffold material is essential to the formation of a proper cellular barrier. It is, therefore, pertinent to divert urine during the first week from implantation of a tissue-engineered graft.

#### 4.2.2. Self-Renewal Capability

Research on stem cell deliver promising approaches in improving healthcare for human beings [55]. Self-renewal capability is the ability of the stem cells to divide in order to make new stem cells and thus expand the stem cell pool. Therefore, a source of stem cells with high multipotent differentiation and self-renewal capacities that can be attained through approaches that are not only simple but also non-invasive is very desirable. UDSCs have the self-renewal capability, which is higher at the initial passages, such as passage 2 or 3 [39] Zhang et al. [31] found that cell subpopulation isolated from urine shares many characteristics with MSCs, like cell growth patterns, expansion capacity, clonogenicity, the expression profile of cell surface marker and multipotent differentiation capacity.

#### 4.2.3. Colony Formation

Kang et al. [36] showed that colony formation showed three times more UDSCs value colony formation in comparison with ADSC. It has also been reported that 5 to 10 colonies of UDSCs can be produced per 100 mL fresh urine [34] and in every 100 mL sample, 6 to 7 clones of USC existed in urine that was collected fresh, 3 to 4 clones of UDSC existed in urine that was preserved for 24 h, and 4 to 5 UDSC clones existed in urine that was preserved for 12 h [39].

#### 4.2.4. Vascularization Ability of UDSCs

Through IL-6 and IL-8 secretion and inhibition of T and B cells and peripheral blood mononuclear cells (PBMNC), UDSCs can produce immunomodulatory effects. IL-6 and IL-8 concentration is higher in UDSC supernatant in contrast to BMSC supernatant when human cytokine release arrays are stimulated. In the future, for the prevention and treatment of diabetic bladder tissue lesions or any other immune system disorder, IL-6 and IL-8 can be the principal immunomodulatory cytokines [31]. Moreover, if the microenvironment is favorable, angiogenic growth factors can be secreted by UDSCs, as VEGF can improve grafted cells’ survival. It can also help in myogenic differentiation and enhance innervations [39].

Zhang D et al. [31] showed that using genetically modified stem cells via VEGF transfection gene considerably promoted myogenic differentiation of UDSCs and induced innervation and angiogenesis. However, adverse side-effects such as hyperemia, hemorrhage, and even death were seen in the animal model due to the viral delivery of VEGF. UDSCs secrete almost 25 types of angiogenic paracrine growth factors that include VEGF, FGF, IGF, HGF, PDGF, and MMP. Angiogenic and immunomodulatory growth factors from UDSCs play a significant role in vascularization [37].

#### 4.2.5. Extracellular Vesicles Secreted by UDSCs

Extracellular vesicles (EVs) include exosomes and microvesicles that have significant beneficial effects in several models of disease [56]. Exosomes from both MSCs and ADSCs have been shown to lessen CNIED (cavernous nerve injury-induced erectile dysfunction) [57,58]. EVs, including microvesicles and exosomes, are critical components in the paracrine secretion of stem cells. Exosome production by human UDSCs can suppress kidney complications in diabetic rats. Moreover, storing and managing EVs is much easier in comparison to stem cells, in order to decrease the chance of formation of a tumor as lower clearance of EVs enhances tumor formation [59]. Prattichizzo et al. proposed that EVs can treat endothelial metabolic memory through the delivery of miRNA [60].

In a study conducted by Ouyang B et al. [59], it was presented that reduce deposition of UDSC-EVs enhanced the improved neurogenic-mediated erectile reaction in diabetic erectile dysfunction and endothelial cell marker expression. The erectile response enhancement was indicated by improved ICP/MAP and ICP ratio, which was attributed to enhanced smooth muscle and endothelium function. 

There are many advantages of exosome-meidated therapy. Exosome faciliates cell-to-cell interaction through the transfer of molecules such as nucleic acid, lipids, and proteins. Since exosomes are nanosized, they can easily penetrate different bio barriers, including the blood-brain barrier. Hence, exosome-mediated therapy provides treatment of earlier distant foci of disease [61].

### 4.3. Markers of UDSCs

UDSCs have expressed MSC markers, as well as other important cell surface markers. In a study conducted by Tayhan SE et al., it has been shown that urine-derived cells (UDCs) contain both stem cells as well as urothelial cells. As a maker of urothelial cells, cytokeratin 7 was used, whereas a negative and positive marker for mesenchymal stem cells of human, CD90, and CD45, respectively, were used [32]. ADSCs and UDSCs have shown a similar positive display of approximately more than 92% for CD73, and CD44 while ADSCs showed higher expression for CD105 and CD90. Immunogenic and hematopoietic markers showed negative manifestation on ADSCs as well as UDSCs [29]. In another study, it was shown that UDCs also exhibit CD44, CD29, CD54, CD90, CD73, CD105, CD166, and CD146, which are surface markers for MSC. UDCs also express markers of embryonic stem cells that include c-Myc, klf4, and Oct4. HLD-DR, CD34, CD11b, CD45, CD14, CD31 and CD19 are markers of hematopoietic stem cells that are not expressed by UDCs [38].

### 4.4. Limitations of UDSCs

Despite many UDSC benefits, there are numerous challenges too [62]. Can adult SCs transdifferentiate in vivo, replenishing the deteriorated tissues, or produce some growth factors which help in the regeneration of tissues of the host? [47] UCs seeded on the side of the lumen of the scaffold are usually washed out through urine, lost during surgery, or ejected mechanically through urethral catheter. Additionally, cells that were successfully retained begin to die within the first week, probably due to inflammation, ischemia, or detachment from the extracellular matrix because of apoptosis [30]. One of the limitations of UDSCs is that urothelial cells cannot be obtained from patients with bladder infection, urethral stricture issues, or trauma. Moreover, urothelial cells in some patients may be affected by bladder stones or some other foreign bodies that can pose challenge to the expansion and isolation of a sufficient population of cells.

The influence of urine on UDSCs after in vitro expansion has not been studied to date. However, considering their source, they are probably more resistant to urine relative to other cell types. Studying the mechanism of this possible resistance would be very beneficial to increase cell surveillance after implantation. Until the layer of urothelial regenerates, stem cells are exposed to urine. It takes a week for the regeneration of urothelium; during that time, seeded cell grafts absorb urine.

The unique challenge in using cells in reconstructive development is whether the cells are robust enough to survive the pressure of the urine and layered structure while retaining functionality. This would result from reciprocal interaction between the cells and the biomaterials used for urinary conduits, and functional studies need to be carried out. However, scaffolds or biomaterials containing urothelial cells and smooth muscle cells differentiated from UDSCs can be promising media for urinary reconstruction [13].

## 5. Urine-Derived Stem Cells in Urethral Regeneration

Urethral reconstruction using cell-seeded methods with autologous cells seeded on biodegradable scaffolds achieves better outcomes in longer segment urethra repair [63,64], compared to non-seeded scaffolds. A suitable cell origin and biomaterial scaffold are both crucial for urethra tissue engineering. Oral mucosa [65] is currently most commonly utilized for urethral tissue engineering.

**Figure 1:**
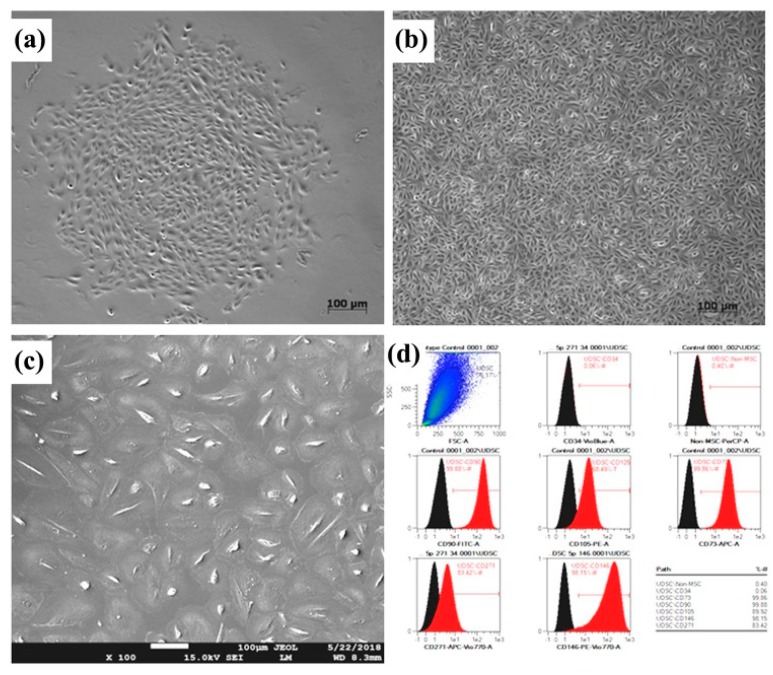
Urine-derived stem cells (UDSC). Isolated primary cells (**a**) and confluent first passage (**b**) colonies. Flattened third passage cells (**c**) contaminated with renal epithelial cells. A representative histogram showing UDSCs positive for mesenchymal stem cells markers, including CD-90, CD-146, CD-73, CD-105, CD-271, and lack of CD14, CD34, CD45, and CD20 endothelial cell expression (**d**). Reproduced with permission from [66].

UDSCs were utilized in several preclinical and human experiments aimed at tissue engineering applications for urethral repair. Urine was collected through urethral catheterization, spontaneous voiding, bladder washing, or bladder irrigation. Cells were cultured in initiation media following centrifugation of the urine samples. Findings yielded indicate that 5−10 UDSC clones/100 mL urine can be collected from majority of freshly voided urine samples [38]. To prepare a cell-seeded biomaterial scaffold for use in urological tissue regeneration, about 50 × 10^6^ cells/cm^3^ must be available for seeding. Thus, a 200 mL urine sample can provide enough cells to create a cell-seeded scaffold 0.5 × 2 × 10 cm^3^ in size (Figure 1).

Tayhan et al. [32] studied both UDSCs and human urothelial cells that were isolated from fresh urine samples after been harvested from six healthy patients via catheterization. Using immunohistochemical assessment, UDSCs reached confluency within 12 days and were positive for cytokeratin 7. Mesenchymal stem cells were also positive for CD90 and CD45. Yang et al. [67] used flow cytometry, immunocytochemistry, and cell proliferation assay to characterize UDSCs harvested from rabbits through either voided urine or bladder washes. Successful differentiation into urothelial, smooth muscle, and osteogenic cell lines was achieved.

**Figure 2:**
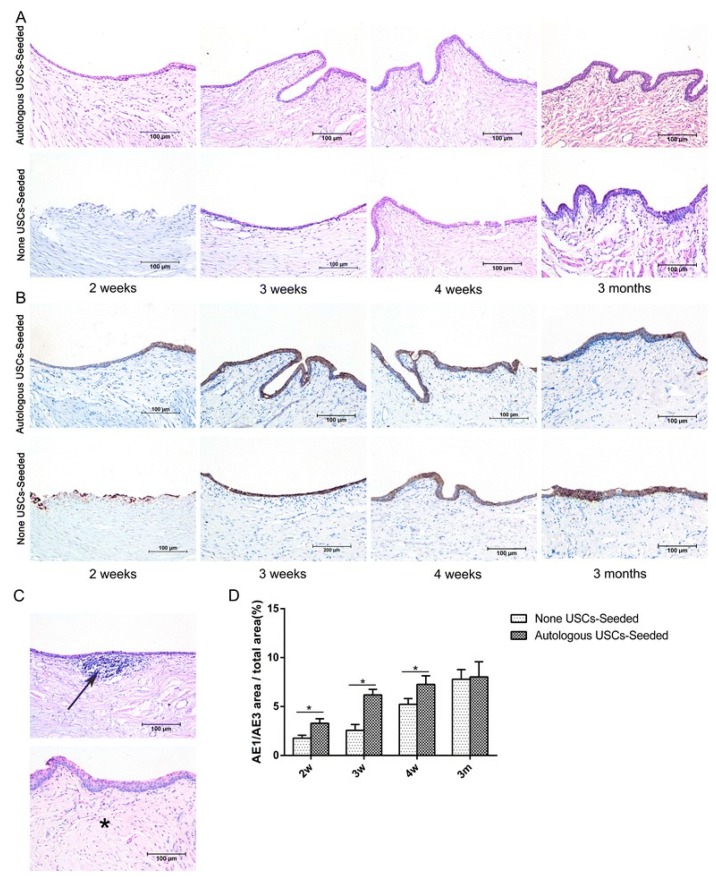
Histopathological evaluation of urothelium regeneration. H&E (**A**) and AE1/AE3 IHC (**B**). The USC-seeded 5% PAA-treated SIS group showed better multilayered urothelial tissue over the different time frames in comparison to 5% PAA-treated SIS only group. (**C**) Infiltration of inflammatory cells (arrow) and fibrosis (*) were observed in the 5% PAA-treated SIS only group. Scale bar = 200 μm. (**D**) Image analysis of the AE1/AE3-positive area to the total area at each time point in the two groups. *Statistically significant (*p* <  0.05). m: months, USC: urine-derived stem cell, w: weeks. Reproduced with permission from [68].

UDSCs from voided urine seeded into small intestinal submucosa (SIS) were tested by Yang et al. and showed differentiation into smooth muscle and urothelial cells. Both static and dynamic culturing approaches were employed by the authors before in vivo implantation. Dynamic conditions resulted in the formation of a multilayered mucosal structure similar to urothelial tissue. [40] On the other hand, autologous rabbit UDSCs harvested via bladder irrigation were labeled with PKH67 and seeded into SIS by Wu et al. [40] Cell-loaded scaffolds implanted into rabbits to repair ventral urethral defect showed differentiation of the cells and regeneration of the urethral defect. [68] (Figure 2)

**Figure 3:**
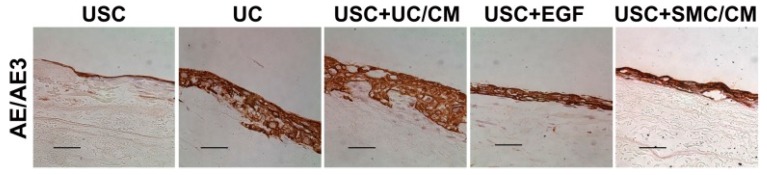
Formation of multilayered urothelium of urothelial cell-induced urine-derived stem cells. Under dynamic culture, the urothelium stained positive for AE1/AE3 like urothelium onto a Small intestinal submucosa (SIS) scaffold. In contrast, USC treated with EGF or SMC/CM produced a thinner layer, and USC alone formed a single layer. Scale bar = 50 μm. Abbreviations: USC = urine-derived stem cells, UC = urothelial cells, SMC = smooth muscle cells, CM = conditioned medium, UC/CM = urothelium conditioned medium, SMC/CM;smooth muscle cell-conditioned medium, EGF;epidermal growth factor. Reproduced with permission from [28].

Yang et al. also seeded UDSCs with dynamic culture on bladder submucosa, which significantly promoted cell-matrix penetration in vitro, as well as cell growth in vivo [69]. Bodin et al. seeded UDSCs on microporous bacterial cellulose (BC), obtaining layered urothelial cells and SMCs with excellent cell-matrix infiltration [55]. Dynamic conditioning was performed on culturing human UDSCs and seeded on SIS formed multilayered uroepithelium in vitro using a Transwell system. Excellent cell differentiation was observed, and the permeability assay confirmed healthy functioning of the urothelial barrier [28] (Figure 3).

## 6. Urine Cytotoxicity

Although somewhat less complex than the urinary bladder, both the urethra and ureters have related structural, functional, and physical characteristics. These tissues are subjected to both radial and fluid shear forces as a result of urine propulsion, transport, and storage. Additionally, these tissues have a lining of epithelial cells called urothelium that guards the underlying tissues against urine, which was recognized to be one of the very significant factors contributing to implanted cell survival when conducting urethral tissue engineering.

Singh and Blandy conducted an experimental study on rats to determine the role of urine extravasation in the urethral stricture pathogenesis [70]. They observed that the ultrastructure of urethral stricture tissue suggested that some strictures were fibrous while others were more resilient, and the total amount of collagen increased in urethral strictures, resulting in dense fibrotic tissue with decreased smooth muscle tissue and decreased elasticity.

Therefore, urine is considered a profoundly cytotoxic agent whose effect in urologic tissue engineering has been undervalued. Studies conducted in vitro on MSCs and urothelial cells cultured in a mixture of urine exhibited tremendous cytotoxicity [71,72]. The recognized cytotoxic impact was not particular for MSC as an experiment showing urine cytotoxic consequences on human urothelial cells was conducted by Davis et al. [71] Those outcomes confirm the central role of urine in the pathogenesis of interstitial cystitis (IC), a condition that manifests as repetitive discomfort in the bladder and the surrounding pelvic area. While the precise root cause of IC has not yet been established, it is known to hinder bladder cell generation and make the healing of cell layers very challenging.

When cells are seeded on the scaffold on the side facing urethra lumen, they are directly exposed to urine, particularly those situated on the inner surface of the biomaterial. Urine is rich in protamine sulfate, products of low molecular weight, and cationic substances that are chiefly responsible for its nonselective and high cytotoxicity. High urea levels, a principal constituent of urine, are associated with a more significant reduction in endothelial progenitor cells (EPCs), a bone-marrow-derived mononuclear cell population that plays a vital role in the preservation of vascular integrity, availability, and function [63]. Recently, Trecherel et al. [73] demonstrated that urea was able to induce the expression of a pro-apoptotic member of the BCL2 family, the Bcl-xL/Bcl-2-associated death promoter (BAD) protein, in VSMC. Likewise, urea was shown to be toxic for HeLa Cells and, in contrast to the initial single wave of arrested mitosis seen with continuous exposure to urea, intermittent exposure resulted in successive peaks of arrested metaphases and had significantly more remarkable effects on the growth and viability of the cultures [74].

In addition, Adamowicz et al. demonstrated that none of the cells seeded on the lumen side of the urinary tract survive beyond 24 h post-transplantation. Although in this experiment, the pH of the medium of cultivation was corrected to 7.4, the urine cytotoxic properties might be faster in a physiological environment when there is an acidic pH (which can be even 5.0). Valuable information was gained from this study when pursuing urinary tract repair using cellular grafts [72]. Therefore, it is critical to implement appropriate urinary diversion when implanting a scaffold within the urinary tract to guard against these unwanted outcomes of urinary leakage into surrounding tissues and allow a healthier healing process to take place.

## 7. Conclusions

Great efforts and significant milestones were achieved in the pathway to attain the maximum benefit of stem cells derived from urine with its inherent fitting characteristics given the ease and limitless nature of this highly versatile fuel source for the cellular component of scaffolds. Still, further studies are needed to better understand and limit the adverse effects of urine on the implanted bioengineered constructs and thus improve subsequent outcomes of tissue-engineered replacements of the urinary tract.

## Figures and Tables

**Table 1:** Comparison of different stem cell types used for urological repair. MSC, mesenchymal stem cell; SMC, smooth muscle cell; UC, urothelial cell; UDSC, urine-derived stem cell. ADSC, adipose-derived stromal cell; BMSC, bone marrow-derived mesenchymal stromal cell; ESC, embryonic stem cell; iPSC, induced pluripotent stem cell; N/A, Not applicable.

Features	Cell Type				
	UDSCs [25,35,38,39,40,41,42]	iPSCs/ESC [43,44]	Bladder SMC, UCs [45,46]	ADSCs [47,48]	BMSCs [23,29,49,50,51]
Harvesting technique	Non-invasive, Low-cost, Easy	Invasive	Invasive	Invasive	Invasive
Stem cell isolation	Very Easy	Easy	N/A	Neutral	Neutral
Angiogenic trophic factors	Yes	Unknown	Limited	Yes	Yes
Immuno-modulatory properties	Yes	Unknown	Unknown	Yes	Yes
Oncogenic potential	No	Yes	No	No	No
Telomerase activity	75% of UDSC have Telomerase Activity	Have Telomerase Activity	No Telomerase Activity	Unknown	Unknown
Endothelial and urothelial differentiation capability	High Ability (60–85%)	Yes	N/A	Yes	Yes
Multi-lineage differentiation capability	Multipotent	Pluri-potent	N/A	Multipotent (mesodermal cell lineages)	Multipotent (mesodermal cell lineages)
Self-renewal capability	High Capability	Strong Capability	Capable	Unknown	Capable
